# When caring becomes an art - how clinical gaze are perceived to be developed

**DOI:** 10.1080/17482631.2022.2156659

**Published:** 2022-12-08

**Authors:** Marie-Louise Södersved Källestedt, Margareta Asp, Anna Letterstål, Margareta Widarsson

**Affiliations:** a Källestedt Clinical Skills Center, Region Västmanland, Västerås, Sweden; b School of Health, Care and Social Welfare, Mälardalen University, Västerås, Sweden; c School of Health, Care and Social Welfare, Mälardalen University, Eskilstuna, Sweden; d Department of Medicine Solna, Karolinska Institutet, Stockholm, Sweden

**Keywords:** Clinical gaze, clinical skills, education, health care, learning, patient safety, professional competence, reflection

## Abstract

**Purpose:**

This qualitative study describes nurses’ experiences and perceptions of how they develop the clinical gaze.

**Methods:**

This qualitative study used an inductive approach and content analysis to assess the experiences of newly graduated nurses, nurse managers, and nursing teachers. Nineteen interviews were conducted. To achieve credibility, the study followed the guidelines of the Consolidated Criteria for Reporting Qualitative research (COREQ).

**Results:**

Two themes emerged: nurses’ personal abilities and the learning culture. Learning culture was considered the foundation of the development of the clinical gaze. The clinical gaze was found to be developed in relationships with patients and when learning together with colleagues, in which the opportunities for reflection are central. To develop the clinical gaze, structures for learning activities, such as reflection, communication exercises, and simulation, are needed so that they become a natural part of daily work. This can also be achieved through supervision and skills training both at university and in a care context.

**Conclusions:**

Prerequisites for the development of the clinical gaze include physical presence with the patient combined with learning activities such as conscious reflection with others in a safe learning culture.

## Introduction

Nurses have close contact with patients and therefore influence patient outcomes and safety (Hendricks et al., 2015; Kvande et al., 2016). Although nurses must be able to plan, organize, prioritize, lead, and evaluate care, contact with patients is often characterized by unpredictability (Larson & Sahlsten, 2016; Wolf et al., 2010). To deliver such nursing care, nurses must develop the clinical gaze as a foundation of their professional competence. Professional competence is related to nurses’ ability to apply their professional and personal qualifications in a given context (Delmar, 2013). The clinical gaze is a complex competence that involves “intuition and the use of a sound, rational, relevant knowledge base in situations that, through experience, are so familiar that the person has learned how to recognize and act on appropriate patterns” (Easen & Wilcockson, 1996, p. 672). Intuition is defined as “understanding without rationale inherent in the expert practice of operating from a deep understanding of the total experience” (Benner & Tanner, 1987, p. 32). This understanding comes from experiences in several situations in which the nurse has seen variations of a specific phenomenon (Marton & Tsui, 2004). Nurses must be encouraged to not focus solely on routines, but to trust their intuition, recognize if anything is abnormal, and act (Berterö, 2010).

The clinical gaze was described in a study of how nurses assess changes in patients’ conditions in intensive care (Kvande et al., 2016). The competence for making such assessment is described as sensitive situational attention and involves being sensitive and emotionally present, systematic, and concentrating, physically close to the bedside, and trained and familiar with the routines. This description was developed in an intensive care context with critically ill or unconscious patients. In other health care settings in which the nurse can communicate with the patient, the clinical gaze focuses on being aware of the patient’s needs but also life phenomena, such as the patient’s experiences of hope, suffering, and anxiety (Delmar, 2013). One way to understand these phenomena has been described by Løgstrup (1995), who states that all understanding is based on emotions and sensations, and sensations provide an access to the world. As part of sensitive situational attention, nurses use their sensations, which can also be understood in the context of Merleau-Ponty’s (1962) ideas about bodily knowledge and the ability to access the world through one’s lived body (Kvande et al., 2016).

A well-developed clinical gaze is required to recognize and act when there is an impaired patient and the patient’s condition changes (Bisholt, 2012; Enger & Andershed, 2018; Kavanagh & Szweda, 2017; Ristić et al., 2021). Every patient and situation provide different challenges as they are both unique and typical at the same time. A sense of the situation and the ability to recognize the unique are prerequisite while, at the same time, the nurse must capture the reality of the situation and act accordingly. The ability to develop professional competence that provides the prerequisites for capturing the specific situation can be described as using theoretical knowledge in combination with professional experiences from similar situations. This is a continuous process that also presupposes personal responsibility and the willingness to learn (Benner, 2001; Delmar, 2013).

Team members in the health care setting who develop the clinical gaze may be aware of patients’ symptoms and needs and may share their experiences within the team (Lachman, 2013). In the health care setting, newly graduated nurses are expected to be competent and ready for work (Masso et al., 2022). However, with highly specialized care, it is impossible to expect that newly graduated nurses can have enough competence for every healthcare situation. Therefore, residency programmes are conducted to meet health-care organizational requirements for newly graduated nurses and to enhance the development of the clinical gaze (Goode et al., 2016; Södersved Källestedt et al., 2020). However, managers within health-care organizations have noted that nursing training does not meet these requirements for developing the knowledge and skills needed for handling acute situations (Södersved Källestedt et al., 2020; Theisen & Sandau, 2013). Newly graduated nurses also describe the feeling of being unprepared because they feel unaccustomed to the culture and working with different colleagues, environment, and routines, as well as their new role as nurses (Gardiner & Sheen, 2016; Keller et al., 2006; Widarsson et al., 2020). Öhlén et al. (2021) highlight the difficulties in synthesizing the different aspects into an integrated understanding of how clinical gaze relates to clinical experiences in nursing.

The characteristics of the clinical gaze have been explained from the philosophical perspective and by empirical studies, and the importance of having a clinical gaze to promote patient safety has been described. However, the process used to develop the clinical gaze and nurses’ experiences and perceptions of how they develop the clinical gaze are not established. Nurses in different positions, such as newly graduated nurses, nurse managers, and nursing teachers, have all experienced how to develop the clinical gaze in close caring situations with patients, and they can contribute to understanding how nurses develop clinical gaze. Understanding nurses’ experiences in developing the clinical gaze will be useful when creating prerequisites for developing the clinical gaze in undergraduate training and further professional development for nurses. The aim of this study was to describe nurses’ experiences and perceptions of how the clinical gaze is developed.

## Methods

### Design

The present study had a qualitative descriptive design and used the inductive approach described by Braun and Clarke (2006, 2022). The current study was part of a larger study of the development of nurses’ professional competence during their training and their first year as graduated nurses from three perspectives: newly graduated nurses, nurse managers, and nursing teachers (Letterstål et al., 2022; Södersved Källestedt et al., 2020; Widarsson et al., 2020). The intention was that interviews with experiences from three different perspectives would enrich how nurses develop professional competence. To achieve credibility, the present study followed the guidelines of the Consolidated Criteria for Reporting Qualitative research (COREQ) (Tong et al., 2007) (see Supplementary Material).

### Settings, participants, and data collection

Data were collected through interviews conducted by all authors using a semi-structured interview guide in 2017 (see Appendix 1). The plan was to interview the newly graduated nurses in groups but, because of their working situations, the interviews were conducted using interviews with individuals, pairs, and groups. The rationale for the different interview forms was to provide a secure interview situation that would also be practical for the participants. Nurse managers were invited to participate in individual or pair interviews because some of the managers had shared leadership. The nursing teachers at the university were chosen strategically with the intention of capturing descriptions from faculty members with different areas of expertise to increase the breadth of the experiences analysed. Because there is no single way to conduct interviews, compliance with the informants’ need to be interviewed individually, in pairs, or in groups was sought (Taylor & de Vocht, 2011).

A total of 29 participants participated in 19 interviews: 27 women and two men from a region in the middle of Sweden participated. We conducted six interviews involving 11 newly graduated nurses who were participating in a clinical introduction programme and had worked as a nurse for 1 year. We conducted six interviews with nine nurse managers employed in healthcare organization who had extensive experience working as a nurse and whose experience as a manager ranged from 2 to 7 years. We also conducted seven interviews with nine nursing teachers at the university, four of whom were lecturers in theoretical courses, three were lecturers responsible for education in clinical settings, and two were leaders of nursing education. Their lecture experience ranged from 5 to 15 years. All participants were nurses with a Bachelor of Science in nursing. All participants are henceforth referred to as “professionals”. To enhance the transferability of the study findings, we recruited participants who were registered nurses with experience from different specializations such as medicine, surgery, and psychiatry in both the adult and paediatric fields.

The interview guide was pilot tested and used in all interviews to enhance the study’s credibility. The interview guide was tested on newly graduated nurses and nurse managers, and no corrections to the interview guide were needed. The interview guide covered the following three areas: integrating theory and practice, the clinical gaze, and competence in leadership. In the present study, the answers to the questions concerning the clinical gaze were analysed. The analysis of the integration of theory with practice and competence in leadership has been presented in other studies (Södersved Källestedt et al., 2020).

To maintain confidentiality, the professionals are referred to here using an abbreviation and number (Table 1). To ensure the trustworthiness of the study, all interviews were audio-recorded and transcribed verbatim by a certified transcriptionist. The interviews lasted 33–99 minutes: newly graduated nurses 62–85 minutes, nurse managers in health care settings 33–59 minutes, and nursing teachers at university 42–99 minutes.

**Table 1.** Overview of participants.

Interview (n=19)	Participants (n=29)	Professional title	Capitals+ Newly Graduated Nurse=NGN Nurse Manager=NMgr Nursing Teacher=NT	Interview time (33-99 min)
1	(n = 2)	Newly Graduated Nurse	NGN1, NGN2	85 min
2	(n = 3)	Newly Graduated Nurse	NGN 3, NGN 4, NGN 5	75 min
3	(n = 2)	Newly Graduated Nurse	NGN 6, NGN 7	75 min
4	(n = 2)	Newly Graduated Nurse	NGN 8, NGN 9	83 min
5	(n = 1)	Newly Graduated Nurse	NGN 10	62 min
6	(n = 1)	Newly Graduated Nurse	NGN 11	72 min
7	(n = 2)	Nurse Manager	NMgr1, NMgr2	59 min
8	(n = 1)	Nurse Manager	NMgr3	33 min
9	(n = 2)	Nurse Manager	NMgr4, NMgr5	49 min
10	(n = 2)	Nurse Manager	NMgr6, NMgr7	41 min
11	(n = 1)	Nurse Manager	NMgr8	49 min
12	(n = 1)	Nurse Manager	NMgr9	44 min
13	(n = 2)	Nursing Teacher	NT1, NT2	55 min
14	(n = 3)	Nursing Teacher	NT3	69 min
15	(n = 2)	Nursing Teacher	NT4, NT5	99 min
16	(n = 2)	Nursing Teacher	NT6	81 min
17	(n = 1)	Nursing Teacher	NT7	42 min
18	(n = 1)	Nursing Teacher	NT8	49 min
19	(n = 2)	Nursing Teacher	NT9	59 min

+ Participants were anonymized by being assigned an abbreviation and a number.

### Data analysis

Data from newly graduated nurses, nurse managers, and nursing teachers were analysed together. The transcribed interviews were analysed using the five phases of thematic analysis of Braun and Clarke (2006, 2022). The coding process was performed using New NVivo (Alfasoft NVivo release 1.3). In phase one, all authors read the transcripts several times to familiarize themselves with the data. Notes and labelling for coding were made with a focus on clinical gaze when something was expressed specifically about the clinical gaze. The authors (MLSK, MA, AL and MW) created codes in NVivo individually. In phase two, they compared their codes and organized the codes into groups with similar meanings, and a mind map was created to visualize the codes. In phase three, the codes were sorted into potential themes and sub-themes. Notes, codes, and themes were constructed inductively according to the data together in the whole research group. In phase four, the themes identified were refined. In phase five, the essence of each theme was generated with the definition and name of the themes. To ensure the credibility of the analysis, all authors read and discussed the content several times during the analysis process.

### Ethical considerations

The present study did not involve patients or sensitive personal information and, therefore, the study does not fall within the boundaries of the Ethical Review Act, 2019:460. But we followed the requirements of the Declaration of Helsinki (World Medical Association, 2014). Permission to conduct the study was given by the chief manager at each health care organization. An assessment was made of the relationship between the value of the project and any potential burden or risk for the participants. Potential participants received both verbal and written information explaining that their participation was voluntary and that they could withdraw from the project at any time. Informed consent was obtained from all participants. To guarantee confidentiality, the presentation of the results ensures that no participant can be identified.

## Results

**Figure 1.**
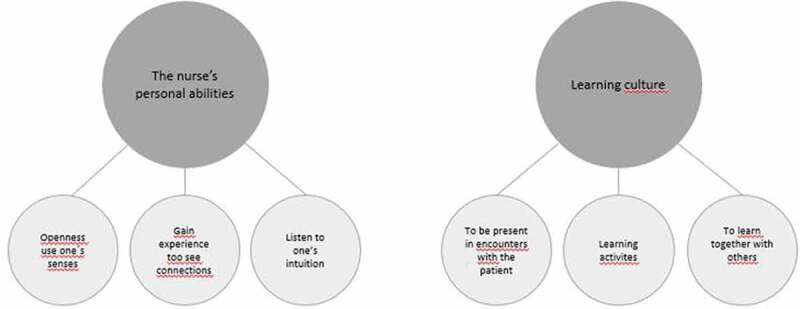
Experiences from newly graduated nurses, nurse managers, and nursing teachers regarding the development of clinical gaze.

The overall results of the analysis of these professionals’ experiences of the development of the clinical gaze are presented in two themes: (1) the nurse’s personal abilities, and (2) the learning culture (see Figure 1). The theme the nurse’s personal abilities contained three sub-themes: openness to use one’s senses, gain experience to see connections, and listen to one’s intuition. The theme the learning culture contained three other sub-themes: to be present in encounters with the patient, learning activities, and to learn together with others.

### The nurse’s personal abilities

The nurse’s personal abilities affect the development of the clinical gaze. Personal ability means the ability to openly use one’s senses by seeing, listening, and feeling, thereby being able to make assessments and prioritize the patient’s needs. The ability to note changes in the patient’s condition is described as complex and difficult at the beginning of professional practice, but this ability develops with experience. The nurse’s personal abilities also include intuition, which is important for the development of clinical gaze.

#### Openness to use one’s senses

Nurses use their acquired knowledge and senses to assess and prioritize the required care measures. The ability of nurses to use their senses and integrate their own knowledge and experiences requires them to be able to see behind the obvious. The development of the clinical gaze was described as, “*You look at skin colour, respiration, even these physical symptoms. But also, to be able to see behind, how do you feel, how is it, to stop, to be present, and to be able to see that part*” (NT8).

When meeting the patient, the nurse prefers to ask open-ended questions to take part in the patient’s subjective experiences while also evaluating the patient’s temperature, skin, elimination, dietary intake, and breathing patterns. The nurse’s clinical gaze is therefore developed while analysing and processing this information and before implementing the appropriate preventive measures.

The clinical gaze also involves developing the ability and feelings to trust their own sensory impressions and to reflect critically on these in relation to objective parameters. One participant described this as, “*How he/she* (the patient) *actually feels, without looking blindly at the papers and numbers and concluding that the patient is unwell. But how does the patient feel in bed? Or, the numbers look good, but the patient doesn’t feel well in bed*” (NMgr2). Participants described the importance of using all the senses to see small changes along with the help of technical equipment as part of developing the clinical gaze by “*hearing, feeling, smelling, in other words smelling, and touching*” (NT5).

During training, nurses learn to use their senses to observe the patient and the care process as a structure through a progression that should help the nurse develop the clinical gaze. The development of the clinical gaze is perceived to take place through years of interactions with different patients, during which nurses use, train, and develop their senses.

The senses are used and trained through different ways of learning; for example, through continuous simulation exercises during training and after graduation that enable “*mind presence and mind training*” (NT6). The development of the clinical gaze takes place by learning to “*assess according to see, listen, feel, A to E structure* [structured assessment of the patient according to the ABCDE method], *to detect failure in vital functions in time*” (NT6). Part of the development of the clinical gaze is to repeat steps to develop the ability to use the senses. One nurse expressed this as, “*So, A to E, I had learned at school. But we got two emergency simulation days here* [at the hospital’s clinical skills training centre] *… and the emergency simulations are always good to rehearse because you can always learn more about that*” (NGN8). In other words, by practising to use one’s senses, one can learn to see what is not visible at first.

#### Gain experience to see connections

Developing the clinical gaze requires the nurse to gain experience through many encounters with patients. Experience is needed for the nurse to be one step ahead of any failure in the patient’s functions. The clinical gaze develops differently in each individual. As one nurse manager said, “*You are different then, as a human being and see different things and think in different ways*” (NMgr7). Developing the clinical gaze is about learning to see the entire situation in context. One participant stated, “*Some have a kind of innate clinical gaze, I think, more than others. Some may know really well theoretically, but they do not see the context*” (NT7). Experience and time are needed to see the connections. One participant expressed this, “*At the beginning when you have no experience, you do not know. So, then everything comes to oneself and then you wonder, what should I do with all this information and how should I sift it and assess it?*” (NGN7).

The newly graduated nurse also needs to care for patients with various conditions to be able to “*relate to more than just the book*” (NGN11). The training was perceived to help them gain experience. One participant said, “*train, feel, and squeeze, and then learn in that way*” (NMgr3). The accumulation of experience begins with work-based training (WBT) and work-integrated learning (WIL) during nurse training and then continues through the care of patients by professional nurses.

#### Listen to one’s intuition

Listening to one’s intuition is a significant part of the development of the clinical gaze whereby the nurse gains a feeling and certainty about the mismatch between how the patient looks and acts in comparison with objective data. This was commented on by several participants. “*But just this gut feeling, the intuition that there is something wrong with this patient when you check … You may [decide to] measure blood pressure or blood sugar and you may measure saturation just because you get a feeling*” (NMgr3). Another participant commented on the application of intuition, how the newly graduated nurse uses intuition to note that something is not right with the patient:

*We removed her catheter, and she had been lying for maybe 2 hours and wanted to get up to urinate. And then I thought at once that she fluttered her eyes very much. It did not feel like … I have no contact with her. She answered speech, but I did not think she was present in her eyes. And then I felt it was not a good idea to make our way to the toilet now. So, I asked her to sit down again, and as soon as she sat down on the bed, she fainted … So, you have to see it* (NGN11).

When assessing a patient’s vital functions, the nurse develops intuition and understanding of when a measure is inconsistent with the patient’s apparent condition. This awareness of a mismatch between readings and the patient’s condition is an important part of the clinical gaze. This clinical gaze is perceived as an asset in nursing.

### The learning culture

Development of the clinical gaze begins during university training and continues in the care context, and both arenas contribute to the development and integration of knowledge. A developed learning culture provides the conditions needed for nurses to develop the clinical gaze. An example is allowing time during training for reflection as part of the learning activities. As the clinical gaze develops in a permissive learning environment, the nurse can move from a task-oriented gaze to seeing and assessing the patient’s entire situation. The patient’s entire situation is not always obvious, and it can be difficult for the inexperienced nurse to detect the entire situation.

#### To be present in encounters with the patient

A learning culture that values the nurse’s presence at the patient’s bedside, seeing the whole person, and assessing their needs is perceived as an important aspect in the development of the clinical gaze. One participant said, “*It is the whole, to see the patient, so you must also practice that*” (NMgr7). The ability to see the overall picture of the patient’s needs takes time to develop. One nurse noted that the focus should be on being able to see the entire situation and described the following conversation between an experienced nurse and a newly graduated nurse.

*You go in* [to the patient] *and look whether she has oxygen* [being delivered to] *her nose. I went in and saw, yes, she did but it had slipped out a bit and I put it back in* [to the nose]. *The experienced nurse asked, “Well, was she blue through the lips?” I answered, “wait a minute, I’ll go back and look”. I had initially looked at the patient and did the specific task requested [check whether the patient was receiving oxygen]. After reflecting after the experienced nurse asked about the patient´s appearance, I realized “my God, I cannot just do one task, but I have to notice more”* (NGN9).

The learning process starts during the training period and continues after graduation when the nurse gains further experience in assessing and caring for patients with different needs. The experience of these participants is that the clinical gaze develops when the nurse can be present with the patient to see the whole of the patient.

The patient’s confirmation can be an aid in seeing what is not obvious. Sometimes nurses must be reminded by colleagues about the importance of talking to the patient. It is also important to have physical contact in encounters with the patient. One participant stated, “*that you feel the patient and that is when you get the best idea of how it really is*” (NGN1). In that case, the clinical gaze develops when the nurse analyses and processes information before implementing the appropriate and preventive measures.

Another participant noted that encounters with the patient are central to the development of the clinical gaze, “*Yes, you need to learn to meet other people. To dare to meet a sick person and develop a caring relationship with that person, and then also do something out of the context of this care relationship*” (NT9). The care and the medical measures support each other, and encounters with the patient are part of the development of the clinical gaze. The clinical gaze develops over time and experience, and integration involves thoughts, feelings, and actions.

#### Learning activities

The types of learning activities are prerequisites to the development of the clinical gaze. Appropriate activities can help the nurse gain an understanding of the whole patient. At university, reflection is introduced as part of teaching and learning in the professional development context. Opportunities are also created for reflection in care contexts during in-service training. However, it does not appear that reflection is a natural learning activity within the collaboration between colleagues in the care context. Development of the clinical gaze requires spontaneous conversations as well as structured opportunities for reflection. This is a conscious reflective approach in which the integration of theory and practice is part of the development of a clinical gaze. One participant noted the following:

*Colleagues and mentors, supervisors, take such an approach to make one think* [They might say] *“When we were with that patient … what did you see?” to get you to describe it yourself in your own words … And they had that look, one thing is also to ask smart questions. Yes, again, this is a lot, this pedagogical approach* (NT7).

However, reflection may be difficult to include in everyday clinical practice because it requires more time with the patient, especially for a newly graduated nurse. One participant stated, “*Many times it took longer because I wanted to ask someone before I did something, is this how I should do it?*” (NGN7). This can lead to the nurse experiencing stress, and thus the clinical gaze becomes too narrow where the overall focus on the patient is lost.

Different learning activities can facilitate and be significant in the development of the clinical gaze because individuals learn in different ways. One participant noted, “*Videotaping and visualization I think are very important … some learn through reading, some by doing, and some by seeing or writing*” (NMgr8). Other learning activities mentioned included simulation training exercises to improve the nurse’s ability to observe and develop practical skills. Systematically structured learning activities were thought to be important for these exercises and especially for emergency situations. One participant said, “*When we got to practice this A to E and the team training and failure in vital functions and things like that … It was good to train the clinical gaze in a structured way. Especially in emergency situations*” (NGN2).

To facilitate the integration of theory and practice in learning activities such to stories about patients, seminars, workshops, and activity-integrated learning, the nurse is encouraged to connect through reflection on patient meetings during the WBT. Being present in the encounters with the patient is about listening to what is said and being aware of what is not expressed in words. One participant stated, “*You are present. You listen to both what is said and what is not said*” (NT8). The nurse’s presence in encounters with the patient is considered important to the development of the clinical gaze.

#### To learn together with others

The participants described the importance of the work climate to the learning culture in the workplace to enable the development of the clinical gaze. “*It is the climate in the department. A permissive climate, where you get to be new, you are there to learn*” (NGN8). Good role models who can convey experience-based knowledge to newly graduated nurses are essential for learning. One participant expressed concern about the lack of role models. “*The problem is that we have no role models to learn from. This silent conveyed knowledge that the artisan part of the nursing profession needs to have and convey, it no longer exists*” (NT6).

Some newly graduated nurses request conversations with experienced nurses but, at the same time, they perceive the risk that the experienced nurses may hinder the newly graduated nurses’ development of the clinical gaze because newly graduated nurses think of themselves as slow and insecure in their performance of care tasks. One participant noted that the learning culture influences the development of the clinical gaze and explained it as follows.

[One] *can feel a little inferior with someone who is a super professional*, [for example] *in handling needles and doing everything quickly. If you are new and insecure, then you can become even more unsafe, then this talented person sits down and says, “What did you see here? Have you thought about this?”* (NT7).

Learning takes place together with colleagues in the same profession and through collaboration with other health professionals. One participant noted, “*Together you learn the clinical gaze because you get help from each other all the time*” (NGN3). Collaboration with assistant nurses is also important to the development of the clinical gaze. Doctors are also mentioned as helping to engage newly graduated nurses by both asking and answering questions. As one nurse described, “*So, I ask the doctors a thousand questions every time I’m on the rounds because I want to know things. ‘What is it and what does it mean?’ Because this helps you to become better*” (NGN3). Learning together presupposes “*developing social skills in terms of teamwork, considering my employees. In the team, and then relatives, are also important*” (NT9). In other words, when developing the clinical gaze, it is important to take a reflective, humble, and open approach in the social interactions with colleagues and to take advantage of the opportunities to ask questions and learn with others.

## Discussion

The results of our study suggest that the clinical gaze develops as part of an ongoing process that combines theoretical knowledge and practical experience and that uses the nurse’s senses and intuition to assess a patient’s needs. Development of the clinical gaze is dependent on a learning culture that values the nurse’s presence at the patient’s bedside and the opportunities to learn together with colleagues, for reflection, and learning activities that allow nurses to integrate their knowledge into professional practice.

Managers of newly graduated nurses have identified the obstacles to developing the clinical gaze. An important obstacle is the limited time for nurses to develop competence in advanced care while creating relationships with their patients (Södersved Källestedt et al., 2020). The mediation between the capacity of nurses and the professional requirements is complex. Previous studies of newly graduated nurses have reported that nurses perceive their inadequacy in developing professional competence. They also perceive that universities and health-care organizations have different views about the competencies important in the nursing profession and of the learning process (Gardiner & Sheen, 2016; Widarsson et al., 2020). These perceptions should be considered when planning programmes to develop the clinical gaze.

### The nurse’s development of the clinical gaze

We found that the nurse’s personal abilities and senses are prerequisite for the development of the clinical gaze. With sensitivity and presence in encounters with the patient, the nurse can become aware of changes and prioritize actions to provide proper care at the right time. However, this awareness must go beyond simply routines. The presence and situation-specific attention must be open to seeing and being aware of the life phenomena and their significance for the patient’s well-being (Delmar, 2013). Life phenomena, such as hopelessness, trust, and suffering, are visible beyond physical needs. Theory and praxis can be intertwined given the nurse’s openness to impressions and sensations in the patient’s lifeworld and the nurse’s conceptual knowledge about life phenomena. These will help to facilitate development of the clinical gaze (Ekebergh et al., 2018; Öhlén et al., 2021).

During nursing training, the nurse may struggle in filtering significant information about the patient and obtaining a sense of the whole situation (Delmar, 2013; Widarsson et al., 2020). In this struggle, training is vital (Ewertsson et al., 2015; Waxman, 2010) and a prerequisite for the development of the nurse’s personal competence. Focusing on sensations can be a departure from understanding (Løgstrup, 1995). Previous research has shown that clinical gaze is an essential part of the nurse’s clinical assessment of patients and that widening the clinical gaze can enrich patient safety (Lachman, 2013; Lamprell & Braithwaite, 2019; Langkjaer et al., 2021).

The participants noted that intuition is a part of the clinical gaze. This means that the nurse can assess the patient’s situation, anticipate events, and prioritize to provide individualized and safe patient care. The clinical gaze can be expressed as the art of care. Nortvedt and Grimen (2006) write that the clinical gaze is about actions as well as assessments and the ethical values, which can take time. They emphasize that intuition is a clinical sensitivity when the nurse is present in thought and vigilant about the patient’s situation in relation to the actions performed. Marton and Tsui (2004) believe that awareness changes dynamically all the time and that each situation is experienced in the light of previous experience. Our findings are consistent with this concept in that we found that development of the clinical gaze is a constant process that requires time and opportunities for reflection in daily work. Our findings are also consistent with the findings of Hurteau et al. (2020) that the development of intuition is fostered by expertise and experiences. Also consistent with the findings of Kvande et al. (2016), our study emphasizes the importance of being physically present with a patient to develop the clinical gaze. This means that nurses must, in a sensitive and attentive way, be close to the patient and be able to use all their senses such as sight, hearing, smell, and touch.

Our results also suggest that experience is essential to developing the clinical gaze and becoming a professional nurse. To gain experience, the nurse must be provided situations to care for many patients with different conditions but, at the same time, the number of patients for each nurse must be adjusted according to the nurse’s experience and capacity. Langkjaer et al. (2021) also believe that the clinical gaze is a competence that develops after gaining experience from previous encounters with patients and may involve many years of training.

### Impact of the learning culture on the development of the clinical gaze

Developing the clinical gaze is an ongoing process that, in addition to clinical experience, requires the opportunities for reflection on a regular basis. Conditions must be created within a learning culture in which reflection is integrated into daily work. Our findings highlight that the clinical gaze is developed in a learning culture in which communication, including reflection and learning, occurs with other health care professionals and patients. Residency programmes are conducted to meet health-care organizations’ requirements for newly graduated nurses and to enhance the learning culture and offer learning activities for the development of the clinical gaze (Goode et al., 2016; Södersved Källestedt et al., 2020). Enger and Andershed (2018) note that nurses need interactions with experienced professionals with greater expertise and more opportunities for training.

We also found that newly graduated nurses seek support from experienced nurses in connection with the assessment of a patient’s status and nursing needs, as noted by Manetti (2019). When experienced colleagues ask questions that enable reflection in connection with assessments, learning is made possible for newly graduated nurses, and this will hopefully allow the nurses to handle similar situations more independently in the future. In this way, the nurse can gradually develop from being inexperienced to becoming more experienced (Benner, 1982). This process also gives nurses the opportunity to identify their own learning needs (Sterner et al., 2019), which is a prerequisite for developing a clinical perspective.

The participants in our study emphasized that students use reflection as a method for deeper learning when at university but not on a regular basis as a newly graduated nurse in clinical practice. Ekebergh et al. (2018) describe reflection as the hub of the interaction between the patient and the nurse, which supports the nurse’s learning and care of the patient. Herbig et al. (2001) suggest that unreflected informal learning can lead to wrong decisions, which may decrease patient safety. Enger and Andershed (2018) claim that good cooperation and communication, ability to report, and expertise in the ward can help to develop the clinical gaze. Allert et al. (2021) argue that good communication and a learning culture that fosters training in teamwork and collaboration are necessary in all workplaces.

To create a learning culture in health-care organizations where continuous development of competence is vital, care activities and following the department’s routines are important. However, it is also important that the nurse is given the opportunities to be present in all encounters with their patients. An important prerequisite for developing the clinical gaze is that the learning environment is strengthened so that continuous development of competence is possible through, for example, simulation training and structured reflection. According to Enger and Andershed (2018) and Willman et al. (2020), this is in agreement with nurses’ demands and highlights that nurses want the opportunities for further training and to be able to consult an experienced colleague.

Hope et al. (2011) emphasize that simulation is a method for learning that facilitates the integration of theoretical and practical knowledge, which we believe is an important part of developing the clinical gaze. Studies have shown that health-care professionals who work clinically and as simulation instructors increasingly use evidence-based guidelines, provide feedback to colleagues, and initiate reflection in daily work by bringing their experiences from simulation training into clinical work and from their experiences in clinical work back to the simulation (Tamas et al., 2019, 2020). Thomas and Isobel (2019) argue that reflective practice groups are a beneficial way to engage busy nurses in reflective practice and that nurses benefit from having dedicated time, place, and space to reflect on their roles and to talk with their colleagues.

### Relevance to clinical practice

There is a need for an overall learning culture that supports the development of structured competence within the health-care organization. It is about creating a work environment that includes enough staff to allow different learning activities to be a natural part of the daily work that is independent of workload. Examples of learning activities are supervision, structured reflection sessions, communication exercises, residency programmes, and simulations. Together, these learning activities should help nurses develop the clinical gaze, which is essential for ensuring patient safety. An overall learning culture is one in which the development of the clinical gaze can take place in lifelong learning from novice to expert nurse.

## Strength and limitations

The strength of the current study is that the development of the clinical gaze is described from three different perspectives: newly graduated nurses, nurse managers, and nursing teachers. As Gubrium et al. (2012) note, it is important to interview participants who have knowledge about the area of focus and can provide their perspectives. Including people who fulfil a variety of social roles can help to provide a diverse range of perspectives in qualitative research. From the pedagogical and philosophical perspectives, the concept of the clinical gaze is described as something central to the nurse’s role, although how the clinical gaze develops is not understood completely. Our study contributes new knowledge from these three perspectives.

One possible limitation is that the newly graduated nurses’ work experience was too brief to allow them to understand how they developed the clinical gaze. We believe that the inclusion of three groups, the diversity of their work experience as a nurse, and their experiences in leadership added a broader perspective on how the clinical gaze is developed. Therefore, we chose to describe how nurses develop the clinical gaze as assessed through interviews to understand the experiences of newly graduated nurses, nurse managers, and nursing teachers.

The interviews covered questions relating to three areas (Appendix 1), and it is difficult to determine whether and how the questions affected the participants and their way of thinking about these areas. Based on our analysis of the rich material obtained here, we believe that the interview areas of focus have enriched each other.

Data were collected using individual, pair, and group interviews, which was intended to broaden the study (O’Hagan et al., 2013). The safety and comfort of participants are important factors during the interview situation (Gubrium et al., 2012). A strength of our study was the option for the participants to choose how they wanted to participate in the interviews. This is a pragmatic matter in that some individuals feel more comfortable in an individual interview than in a group interview, and these methods complement each other (Kaplowitz & Hoehn, 2001). However, as group interviews aim to generate discussion and negotiation on a topic, whereas individual interviews aim for in-depth probing, it may be problematic to view the data collected by the methods as homogeneous. Nevertheless, the results from the different types of interviews revealed similar content and did not contradict each other.

As Gubrium et al. (2012) note, statements from participants must be evaluated in accordance with the research question and whether the statements capture the experiences of the whole or focus on one participant’s experience. No deeper information about participants’ characteristics was collected beyond their experience, age, and gender, which limits the transferability. By using the same semi-structured interview guide, we were able to maintain stringency throughout all interviews, which enhances the study’s trustworthiness. Themes and sub-themes were discussed by all authors to achieve consensus. A strength of this discussion was that the authors have experience in training, leadership, and clinical work. The results are in large part transferable to other contexts with a similar training system and health care organizations.

## Conclusion

The clinical gaze is fundamental in the nurse’s professional practice. Prerequisites for the development of clinical gaze include the physical presence with the patient combined with systematic reflection with colleagues in a safe learning culture. These experiences in a nursing context are built over time and lead to the development of the clinical gaze. The results from our study provide information for universities as well as health-care organizations about the prerequisites that facilitate the development of the clinical gaze in the form of various learning activities. Future studies are needed to identify a clearer definition of the concept of the clinical gaze in the context of the nursing profession.

## Appendix 1. Interview guide

**The questions were adapted according to the participants in the interview**: **newly graduated nurses, nurse managers, and nursing teachers.**

## Demographics

- What is your profession?
- What is your working profession now?
- Gender

### Opening question

What experiences do you have regarding newly graduated nurses?

What experience do you as a newly graduated nurse have about the transition from formal education at the university to working in a health-care organization?

How do you view the clinical introduction program?

### Focus areas and potential probe questions

(Probes to be used if the area of interest is not covered by the informant’s spontaneous answer to the opening question.)

**Area 1**: **Integrate theory and practice**

- Describe the opportunities to integrate theory and practice during your education.
- Describe the opportunities for integrating theory and practice in the clinical introduction programme. What is your experience of integrating theory and practice from university to working life?
- In your opinion, what can newly graduated nurses do? What is it that they/you have not mastered?
- What are your thoughts on the fact that the nursing that nurses plan for and perform should be evidence based?
- How do prospective nurses learn practical skills?

**Area 2**: **Developing the clinical gaze**

- How does the clinical gaze develop in nurses? In which areas (patient safety, skills, leadership) is development of the clinical gaze most clearly seen?
- What does the clinical gaze mean?
- What opportunities are there for the continuous development of competence for nurses and how do you ensure that you continue to develop your skills?
- How has your experience of security in the professional role changed during the year? (Question asked of newly graduated nurses working for 1 year.)
- How do you ensure that your nurses develop the skills needed to provide proficient and safe patient care? (Question asked of managers.)

**Area 3**: **Competence in leadership**

- What does the leadership function mean for nurses’ work?
- Describe how nursing school and/or the clinical introduction programme provides newly graduated nurses the knowledge and experience necessary to pursue a leadership role. What has facilitated and what has hindered this learning?
- How do you view the collaboration between the county council and the university?
- What measures would strengthen the county council as an attractive employer?
